# Determination of the Accuracy of Salivary Biomarkers for Periodontal Diagnosis

**DOI:** 10.3390/diagnostics12102485

**Published:** 2022-10-14

**Authors:** Hiba Abdullah Mohammed, Ali Abbas Abdulkareem, Faraedon Mostafa Zardawi, Sarhang Sarwat Gul

**Affiliations:** 1 Al-Sadr Specialized Dental Center, Ministry of Health, Baghdad 10011, Iraq; 2 Department of Periodontics, College of Dentistry, University of Baghdad, Baghdad 10011, Iraq; 3 Department of Periodontics, College of Dentistry, University of Sulaimani, Sulaymaniyah 46001, Iraq; 4 Faculty of Dentistry, Qaiwan International University, Sulaymaniyah 46001, Iraq

**Keywords:** biomarkers, saliva, matrix metalloproteinases, periodontitis, tissue inhibitor of metalloproteinases

## Abstract

Background: We aimed to investigate the accuracy of salivary matrix metalloproteinases (MMP)-8 and -9, and tissue inhibitor of metalloproteinase (TIMP)-1 in diagnosing periodontitis and in distinguishing periodontitis stages (S)1 to S3. Methods: This study was a case–control study that included patients with periodontitis S1 to S3 and subjects with healthy periodontia (controls). Saliva was collected, and then, clinical parameters were recorded, including plaque index, bleeding on probing, probing pocket depth, and clinical attachment level. Diagnosis was confirmed by assessing the alveolar bone level using radiography. Salivary biomarkers were assayed using an enzyme-linked immunosorbent assay. Results: A total of 45 patients (15 for each stage) and 18 healthy subjects as controls were included. The levels of all salivary biomarkers and clinical parameters were significantly higher in periodontitis subjects than in the controls. The ROC curve showed that MMP-8, MMP-9, TIMP-1, MMP-8/TIMP-1, and MMP-9/TIMP-1 had statistically significant diagnostic accuracy, with areas under the curve (AUCs) of 0.892, 0.844, 0.920, 0.986, and 1.000, respectively, when distinguishing periodontitis from the controls. Similarly, these biomarkers showed significant diagnostic accuracy in the differentiation of S1 periodontitis from the controls (AUC range from 0.902 to 1.000). Conclusions: This study suggested that salivary biomarkers exhibited high diagnostic accuracy in distinguishing periodontal health from periodontitis in general as well as S1 periodontitis. Furthermore, TIMP-1 could differentiate S1 from S3.

## 1. Introduction

Periodontitis is an immune-mediated disease initiated and progressed by dysbiosis of subgingival microbiota in a susceptible host that causes irreversible damages to tooth supporting structures and tooth loss [1]. Worldwide, the prevalence of periodontitis ranges from 20% to 50% of the population [2] and silently imposes economic, health, quality of life, psychological, and functional burdens [3,4,5,6]. Early diagnosis plays a great role in preventing deleterious consequences of periodontitis [7]. Unfortunately, periodontitis may not be detected at early stages or may be neglected by patients as the disease is painless and there is a lack of awareness in affected subjects on the nature and negative impacts of the disease [8].

Globally, the use of a periodontal probe is considered a gold standard for periodontal examination. However, collecting six site-based full-mouth periodontal charts of clinical parameters such as bleeding upon probing (BOP), probing pocket depth (PPD), clinical attachment loss (CAL), and radiographic bone loss is a relatively long and tedious process and can only provide information about previous tissue destruction [9]. It does not provide information about the current disease activity or its future progression and the likely response to treatment. In addition, periodontal probing could be associated with a certain degree of inconsistency among dentists due to differences in probing force, type and dimensions of the probe, angulation, operators’ skill, and topography of periodontal pockets [10]. Consequently, precise diagnosis could be a challenging task for dentists, resulting in overlapping of periodontitis stage determination.

Over recent decades, new and improved periodontal probes have been introduced, such as pressure-sensitive and electronic probes [11]. Nevertheless, these modifications could not overcome related problems such as inter-examiner variations, the considerable time consumption, and the relatively high cost of some of these tools [12].

The new classification scheme of periodontal diseases and conditions [13] solves many issues related to the older classification system [14]. One of the major changes is adding a case definition of healthy status [15]. In addition, periodontitis is described in multi-dimensional terms, including severity, which is expressed as “stage” (S) and is measured by the level of alveolar bone at a site showing the worst clinical attachment loss (CAL) interdentally [16]. According to the 2017 classification of periodontal diseases, radiographs are required to confirm staging by assessing the alveolar bone level. However, the decision to use radiographs is generally dependent on the findings of the clinical diagnosis [9]. Moreover, there are some limitations with using radiography such as exposure of the patient to ionizing radiation even with the most advanced equipment [12]. On the other hand, radiography simply converts 3D objects into 2D images, which results in underestimation of the disease’s severity and superimposition by other structures [17]. Additionally, the costs of modern 3D radiographic machines such as CBCT are high, and these machines are not available in every dental clinic. Lastly, radiographs only provide an idea about hard tissues and cannot reflect the soft tissue status, which is integral in framing diagnosis statements [12].

These aforementioned limitations of clinical diagnostic techniques (periodontal probes and radiographs) could create issues during diagnosis and the determination of periodontitis staging. It is important to acknowledge that the new classification scheme is designed to incorporate biomarkers in light of future developments. Recently, a point-of-care test has been suggested to be incorporated in the new classification scheme, and the authors claimed the usefulness of this test as an adjunctive diagnostic tool in determining the stages of periodontitis [18].

The use of biomarkers in oral fluids for the diagnosis of periodontal disease has gained popularity in recent decades. Among these oral fluids, saliva is an attractive tool as it is abundantly available and can be non-invasively collected [19]. Matrix metalloproteinases (MMPs), e.g., MMP-8 and -9, are proteolytic enzymes involved in periodontal tissue remodeling during health and are responsible for tissue destruction during disease [20]. The levels of MMPs in oral fluids show reliability in diagnosing and predicting the prognosis of periodontal disease [18,21,22,23]. The proteolytic action of MMPs is neutralized by the tissue inhibitor of metalloproteinase (TIMP)-1, which has the highest levels during health and decreases in association with periodontitis and its level is inversely proportioned with the level of MMPs [24]. Although individual biomarkers show promising results in differentiating periodontal health and disease, the accuracy could be compromised by local and systemic factors [25], which could, nevertheless, be addressed by the use of combinations of different biomarkers [26,27]. Based on available evidence, this study aimed to examine the ability of salivary MMP-8, MMP-9, and TIMP-1, individually or in combination, to diagnose periodontitis and to discriminate periodontitis S1 to S3.

## 2. Materials and Methods

### 2.1. Study Design

This study was a case–control study in which patients with periodontitis represented the cases while controls were subjects with healthy periodontia. The study was conducted at the Department of Periodontics, Al- Sadr Specialized Dental Center in Baghdad, Iraq, from February to August 2021. All procedures were conducted in accordance with the Helsinki declaration and its later amendments for human research. Before enrollment in the study, each patient received full information about the nature and aims of the study before signing an informed consent form. The protocol was approved by the Ethics Committee, College of Dentistry, University of Sulaimani (Ref. #53/21 on 25 October 2021). This clinical study followed the *Strengthening the Reporting of Observational studies in Epidemiology* (STROBE) in terms of study design and reporting of results.

### 2.2. Study Population

The subjects enrolled in this study were patients referred for or seeking periodontal therapy. After recording their demographic data, unstimulated salivary samples were collected from each patient, followed by clinical and radiographic examinations. The periodontitis subjects were diagnosed clinically at first for preliminary determination of the staging; then, the diagnosis was confirmed by measuring the radiographical bone loss of the most affected tooth.

### 2.3. Inclusion Criteria

Subjects recruited in this study were systemically healthy, had a minimum of 20 teeth, and had not been under any medications in the last 3 months. The control group included subjects with BOP < 10%, PPD ≤ 3 mm, and intact periodontium and visited the Al-Sadr Specialized Dental Center for regular checkup [15]. The case definition of periodontitis followed the 2017 classification of periodontal diseases and conditions [16], whereby interdental CAL was interdentally detected at ≥2 non-adjacent teeth or CAL ≥ 3 mm was detected facially/orally associated with PPD > 3 mm at ≥2 teeth. Accordingly, periodontitis patients were divided into three groups: S1 (10-15% bone loss), S2 (bone loss involving coronal 1/3 of the root), and S3 (bone loss involving mid 1/3 of the root). In addition, all periodontitis subjects had generalized extent and distribution (>30% of teeth involved) with grade B or C and unstable status (PPD ≥ 6 mm or PPD > 4 mm with BOP).

### 2.4. Exclusion Criteria

Exclusion criteria included individuals with dental implant(s); suffering from systemic conditions such as liver and/or kidney dysfunction, inflammatory bowel disease (i.e., Crohn’s disease), or diabetes mellitus; with previous history of organ transplant or cancer therapy; had any cardiovascular or renovascular disease/disorder; or were smokers. Additional exclusion criteria included any previous periodontal therapy in the last three months or currently under active periodontal treatment. Patients receiving antibiotic treatment or immunosuppressant medication within the last three months, and pregnant or lactating mothers, were excluded as well.

### 2.5. Periodontal Parameters

Wisdom teeth were excluded from the examination. The clinical parameters were measured for all existing dentition, including full mouth plaque index (PI) [28], full mouth BOP [29], PPD, and CAL. In detail, PI and BOP were recorded as present (1) or absent (0). For PI, the disclosing agent (Biofilm Discloser, Optident Ltd., Ilkley, UK) was used to detect presence/absence of dental plaque. PPD was measured from the gingival margin to the base of the pocket, while CAL was represented by the distance from the cemento-enamel junction (CEJ) to the base of the pocket/sulcus. Full mouth examination was performed using a periodontal probe (Michigan O probe with increments at 1, 2, 3, 5, 7, 8, 9, and 10 mm) at six sites per tooth, namely mesiobuccal, midbuccal, distobuccal, mesiolingual, midlingual, and distolingual.

Radiographic imaging of the tooth most affected by periodontal disease (the tooth with the worst interdental CAL) was selected to confirm the diagnosis. For this purpose, a parallel technique was used with the aid of a film holder to standardize the procedure.

### 2.6. Calibration and Outcome Measures

Calibration sessions for periodontal parameters were conducted on four periodontitis subjects not included in the study, with intraclass correlation coefficients for continuous data of >0.92 and kappa coefficients for dichotomous data of >85%, which were considered acceptable, as previously described [30].

The clinical parameters (PI, BOP, PPD, and CAL) represented the primary outcomes of the study, whereas concentrations of salivary biomarkers were considered a secondary outcome.

### 2.7. Salivary Sample Collection and Analysis

Patients were advised to perform their routine oral hygiene measures. Salivary samples were collected from the study subjects between 09:00 am and 11:00 am before oral examination. The passive saliva drooling method [31] was used to collect the whole saliva in a sterile plastic cup. A micropipette was used to aspirate a measured volume of saliva of 500 μL into a plastic Eppendorf tube with a premeasured 50 μL protease inhibitor solution. Following the collection, samples were centrifuged (Thermo Scientific, Pico 17, Thermo Scientific, Waltham, MA, USA) at 4000× *g* rpm for 3 min and stored at −30 °C.

The concentration of the protein level for each biomarker was measured by enzyme-linked immunosorbent assay (ELISA). Commercially available ELISA kits (all purchased from MyBioSource, San Diego, CA, USA) for MMP-8, MMP-9, and TIMP-1 were used for determining the biomarker levels in the saliva according to the manufacturer’s instructions. The absorbance of all proteins was measured using a spectrophotometer plate reader (Promega, GloMax, Madison, WI, USA).

### 2.8. Pilot Study and Sample Size Calculation

A pilot study was conducted using the first four samples collected from each group. In total, sixteen samples were analyzed in the laboratory by ELISA. The concentration of one of the biomarkers (MMP-8) obtained from a pilot study was assigned to calculate the sample size according to the following formula [32]:

Sample size = r + 1/r × (SD)2 × (Zβ + Zα/2)2/d2

where r (ratio of cases to controls) is 2; SD is the standard deviation (4863.5); Zβ is the standard normal variate for power of 90%, which is 1.28; Zα/2 is a 5% type 1 error, which is 1.96; and d is the expected mean difference between cases and controls (2974.6 pg/mL).

The calculated sample size for the periodontitis group was 42, which was rounded to 45 to avoid drop out of the sample. Accordingly, each periodontitis subgroup (S1, S2, and S3) received 15 patients.

### 2.9. Statistical Analysis

For descriptive statistics, all data were expressed as frequency, percent, mean, standard deviation, and median. Prior to inferential analysis, Gaussian distribution of the data was determined by using the Shapiro–Wilk test which indicated that data obtained from ELISA were not normally distributed. Therefore, comparisons of multiple groups were performed by the Kruskal–Wallis test, and in case of significant results, further intergroup comparison was carried out by using the Bonferroni post hoc test. As there were strong correlations between MMPs and TIMP, the ratios MMP-8/TIMP-1 as well as MMP-9/TIMP-1 were used to examine the accuracy of these combinations in periodontitis diagnosis and in the differentiation of S1 to S3 periodontitis. The periodontal parameters showed normal distributions; thus, a multigroup comparison was conducted using the ANOVA test followed by the Tukey post hoc test when results were significant. Sensitivity and specificity of the biomarkers were determined using a receiver operating characteristic (ROC) curve and the area under the curve (AUC). After adjustment, the level of significance was set at *p* < 0.03 for multigroup comparisons. All statistical analyses were conducted by using GraphPad Prism software (version 9, GraphPad Software, San Diego, CA, USA).

## 3. Results

### 3.1. Study Population

**Figure 1:**
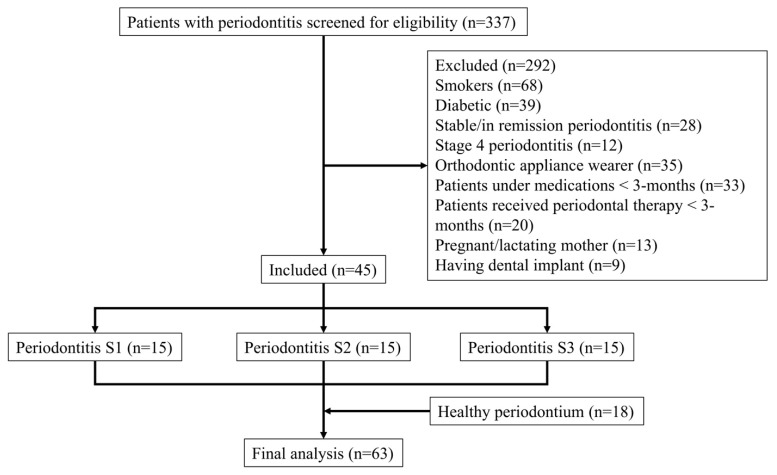
Flowchart of the study. S: stage.

Patients with periodontitis referred for periodontal therapy were initially screened (n = 337) to assess their eligibility for recruitment. After applying inclusion/exclusion criteria, a total of 292 patients were excluded and 45 patients were included in the final analysis. Later, subjects with healthy periodontium and on regular checkup were included (n = 18) as controls (Figure 1). Demographic characteristics of the study population are illustrated in Table 1.

### 3.2. Periodontal Parameters

**Figure 2:**
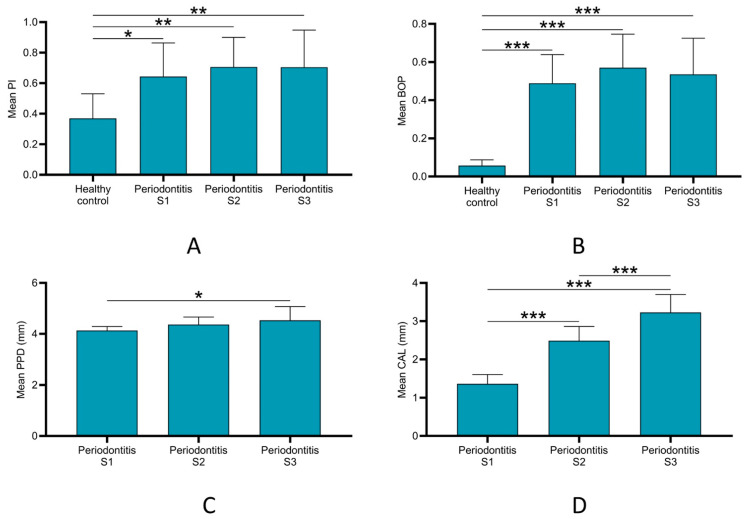
Comparison of clinical parameters among all groups. (**A**) Mean plaque index (PI): periodontitis (S1 to S3) groups exhibited significantly higher PI than control group. (**B**) Mean bleeding on probing (BOP) scores: periodontitis (S1 to S3) groups exhibited significantly higher BOP than control group. (**C**) Mean probing pocket depth (PPD): periodontitis S3 showed significantly higher PPD than periodontitis S1 group. (**D**) Mean clinical attachment loss (CAL): periodontitis S3 showed significantly higher CAL than periodontitis S1 and S2 groups. CAL of periodontitis S2 was significantly higher than in periodontitis S1 group. * *p* < 0.03, ** *p* < 0.01, *** *p* < 0.001. S: stage.

Mean PI and BOP were significantly higher in all stages of periodontitis as compared to the control group. However, no significant differences were detected between the different stages of the periodontitis groups (Figure 2A,B). For PPD, only the periodontitis S3 group showed significantly higher mean PPD as compared to the periodontitis S1 group (*p* < 0.05) (Figure 2C). On the other hand, the periodontitis S3 group exhibited significantly higher (*p* < 0.001) mean CAL than the periodontitis S1 and S2 groups. The latter group also showed significantly higher (*p* < 0.001) CAL than the periodontitis S1 group (Figure 2D).

### 3.3. Salivary Biomarkers Levels

The individual values for each salivary biomarker were determined. Table 2 demonstrates comparisons of the salivary biomarkers’ (MMP-8, MMP-9, and TIMP-1) concentrations and ratios (MMP-8/TIMP-1 and MMP-9/TIMP-1) among different studied groups. Concentrations of salivary MMP-8 and MMP-9 were significantly higher in subjects with periodontitis (S1 to S3) as compared to the healthy control. Meanwhile, the level of TIMP-1 was significantly lower in the periodontitis S2 and S3 groups in comparison with the control group. However, when comparing the ratios of MMP-8/TIMP-1 and MMP-9/TIMP-1, the results showed significant differences in all periodontitis groups in comparison to the control group. No significant differences were observed in concentrations of salivary biomarkers among different stages of periodontitis.

### 3.4. Diagnostic Accuracy of Salivary Biomarkers

Diagnostic potential (sensitivity and specificity) of each biomarker to differentiate between periodontal health and periodontitis and to discriminate different stages of periodontitis was estimated using ROC. All tested biomarkers (MMP-8, MMP-9, and TIMP-1) and ratios of MMP-8/TIMP-1 and MMP-9/TIMP-1 showed statistically significant diagnostic accuracy in differentiating between periodontal health and periodontitis (Table 3).

**Figure 3:**
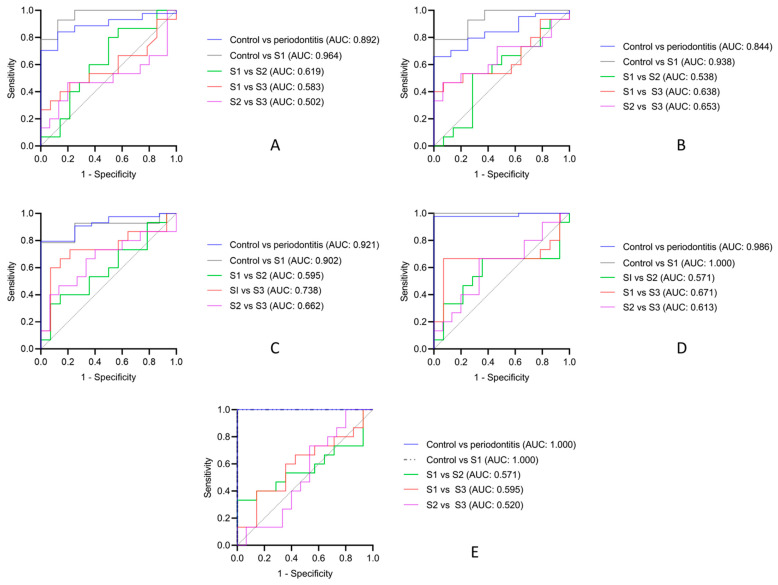
ROC curves for different salivary biomarkers. (**A**) MMP-8, (**B**) MMP-9, (**C**) TIMP-1, (**D**) MMP-8/TIMP-1, and (**E**) MMP-9/TIMP-1. All biomarkers exhibited high sensitivity in distinguishing periodontal health from periodontitis and periodontitis stage (S)1. No biomarker could differentiate different stages of periodontitis, except TIMP-1, which discriminated S1 from S3.

Figure 3 shows the ROC analyses for all biomarkers and their combinations. In summary, the ROC analyses showed that the AUCs for salivary MMP-8, MMP-9, and TIMP-1 were 0.892, 0.844, and 0.920, respectively, between periodontal health and periodontitis. Meanwhile, the AUCs between periodontal health and periodontitis S1 for MMP-8, MMP-9, and TIMP-1 were 0.964 (*p* value = 0.0001), 0.938 (*p* value = 0.001), and 0.902 (*p* value = 0.002), respectively. None of the aforementioned biomarkers and ratios showed statistically significant diagnostic accuracy in differentiation between different stages of periodontitis, except for TIMP-1 in differentiation of periodontitis S1 from S3 (AUC 0.738, *p* value = 0.029) (Table 3).

Interestingly, when using ratios, the diagnostic potential increased with MMP-8/TIMP-1 (AUC of 0.986, *p* value = 0.0001) and MMP-9/TIMP-1 (AUC of 1.000, *p* value = 0.0001) between periodontal health and periodontitis. Likewise, the ROC curve showed a statistically significant diagnostic accuracy (sensitivity and specificity) with an AUC of 1.000 (*p* value = 0.0001) for both the MMP-8/TIMP-1 and MMP-9/TIMP-1 combinations when comparing periodontal health and periodontitis S1. Again, these ratios failed to differentiate between different stages of periodontitis (Table 3).

Based on the results of the ROC curve, biomarkers with the highest diagnostic accuracy in discriminating periodontal health and periodontitis and different stages of periodontitis were subjected to further analysis to determine the sensitivity, specificity, and potential cut-off points (Table 3). The highest sensitivity and specificity (1.000 each) were observed in association with MMP-9/TIMP-1, with a cut-off value of 0.712. This was followed by MMP-8/TIMP-1 (sensitivity of 0.977 and specificity of 1.000), with a cut-off point of 0.464 when comparing control to periodontitis. Similarly, these cut-off points of both MMP-8/TIMP-1 and MMP-9/TIMP-1 combinations showed the highest sensitivity and specificity (1.000 each) for differentiation between control and S1 periodontitis.

All other biomarkers also showed high sensitivity, ranging from 0.786 to 0.977, and specificity, ranging from 0.750 to 1.000, in the differentiation of control vs. periodontitis and control vs. S1 periodontitis.

## 4. Discussion

In the current study, selected salivary biomarkers (MMP-8, MMP-9, and TIMP-1) showed high sensitivity to discriminating periodontitis from periodontal health when used alone or in combination. However, they failed to differentiate periodontitis S1 to S3, except for TIMP-1, which exhibited a potential to discriminate between periodontitis S1 and S3.

Indeed, introducing biomarkers for diagnosing periodontal disease can be of a great value in clinical practice. Therefore, this study attempted to explore the diagnostic potentials of selected salivary biomarkers to screen periodontitis and to differentiate stages of periodontitis, which to the best of our knowledge, has received limited attention.

We followed the latest case definition and diagnostic scheme of periodontal diseases and conditions [13,16], which has introduced revolutionary changes from the previous classification [14]. However, discrimination between stages could be a challenging task for dental practitioners who could find themselves in a grey zone when conventional clinical parameters are used. Additionally, adding further components, e.g., staging, which require meticulous clinical examinations could be an extra burden for the patient and the dentist. Furthermore, conventional clinical diagnostic methods are known to be associated with certain drawbacks [9,10].

Among available oral fluids, saliva is the most preferable both in research and clinical settings. This is due to the collection process being easy, non-invasive, and salivary elements being able to accurately reflect the state of periodontal health and disease [19]. However, it does not provide site-specific information, and individual variations in the salivary flow rate could affect the results. Passive drooling was selected to collect saliva in this study as this method potentially minimizes bacterial contamination of the sample and other unsystematic errors associated with other collection techniques [33,34]. Additionally, a sufficient volume of saliva can be collected in a relatively short period when the drooling method is used [31].

Both MMP-8 and MMP-9 showed high diagnostic accuracy in differentiating periodontal health and disease (AUCs of 0.894 and 0.844, respectively), which is commensurate with other study [18]. MMPs are host-derived proteolytic enzymes that are mainly secreted by polymorphonuclear leukocytes, which are highly increased during periodontal inflammation concomitant with increased levels of secreted MMPs to oral fluids such as gingival crevicular fluid and saliva [35]. This could explain why these biomarkers clearly defined periodontal health and disease but could not differentiate different stages of periodontitis, as in the diseased condition, PMNs are already available in high numbers. In fact, the levels of different MMPs are upregulated in response to gingival inflammation regardless of PPD. Consequently, the levels of MMPs would be elevated in all stages of periodontitis to an extent that cut-off values would be overlapped. The results from the current study were inconsistent with those of another study which recommended the use of MMP-8 in the staging and grading of periodontitis [18]. This could be attributed to the latter study using an active form of MMP-8, using a different assaying technique, and screening a larger sample.

The downregulation of TIMP-1 is associated with the destructive aspects of periodontal disease and has shown potential to diagnose periodontitis [26,36]. Our results were in line with these findings, which supported the use of salivary TIMP-1 as a reliable candidate to diagnose periodontitis. In addition, in contrast to other biomarkers, TIMP-1 differentiated periodontitis S1 and S3 (AUC: 0.738).

Using ratios of MMP-8 and -9 with TIMP-1 favorably increased the accuracy, with AUCs of 0.986 and 1.000, respectively, to distinguish periodontal health from periodontitis as compared to the level of accuracy when each biomarker was used alone. The logic for selecting these combinations was based on the fact that TIMP-1 is the natural neutralizer for MMPs and their levels change in opposite directions during health and disease [24]. When biomarkers are combined, one of them could compensate for the fluctuations in the other ones, thereby increasing the accuracy of diagnosis. These results were consistent with other findings and suggestive of the superiority of using a biomarkers profile instead of a sole biomarker for diagnostic/prognostic purposes [26,27,37]. However, this advantage seems to be limited once periodontitis is initiated, as these combinations failed to differentiate periodontitis S1 to S3. This could be attributed to the upregulation of MMPs to high levels as the disease process is triggered regardless of staging and this classification is based on clinical parameters rather than biochemical parameters.

Periodontitis S1 indicates incipient loss of alveolar bone, which could be difficult to diagnose clinically. Therefore, the accuracy of salivary biomarkers in differentiating between periodontal health and periodontitis S1 was investigated. All biomarkers showed high sensitivity (AUCs from 0.902 to 1.000) in differentiating S1 periodontitis from health. This finding could offer a solution to avoiding a misdiagnosis of periodontitis S1 in clinical practice.

Certain limitations were associated with the current study, mainly the small sample size, which could be comprehended based on the pilot design to preliminarily answer the research question and to establish a foundation for larger-scale studies. Furthermore, results from observational studies only provide an association, not causality, which must be investigated by controlled trials. Ideally, salivary biomarkers should accurately distinguish periodontal health/disease of the whole population regardless of their condition or the presence of risk factors. In this study, only the systemically healthy and non-smokers were included. Nonetheless, this study is one of the few studies that incorporate biomarkers from saliva in order to discriminate different stages of periodontitis. In addition, salivary biomarkers showed a good potential in diagnosing periodontitis. However, it is advised to not generalize these results until they are further confirmed by higher evidence-based trials.

Finally, it would be of great value to investigate the relationship amongst the selected biomarkers and disease onset, the transition from gingivitis to periodontitis, or whether the patient is undergoing active destruction. These are avenues that further studies should explore to improve diagnosis and prognosis.

## 5. Conclusions

Salivary biomarkers (MMP-8, MMP-9, and TIMP-1) exhibited a high diagnostic accuracy in discriminating between periodontal health from periodontitis in general and S1 periodontitis. This accuracy was further increased when combinations (MMPs/TIMP-1) were used. However, generally, these biomarkers could not offer similar diagnostic potential in differentiating periodontitis S1 to S3.

## Figures and Tables

**Table 1:** Demographic characteristics of the study population.

	All Groups	Healthy Control	Periodontitis S1	Periodontitis S2	Periodontitis S3
n, %	63, 100	18, 28.6	15, 23.8	15, 23.8	15, 23.8
Age (years)					
Mean ± SD	29.75 ± 10.93	28.75 ± 4.464	22.00 ± 6.803	28.40 ± 10.55	39.40 ± 10.46
Median	28	28.50	31	34	40
Range	18 to 55	18 to 35	23 to 35	26 to 52	25 to 55
Sex ^§^					
Male	31, 49.1	8, 44.4%	6, 40.0	7, 46.7	10, 66.7
Female	32, 50.9	10, 55.6%	9, 60.0	8, 53.3	5, 33.3

^§^ Frequency, percent, S: stage.

**Table 2:** Comparison of salivary biomarkers levels among study groups.

Salivary Biomarkers ^§^	Control	Periodontitis S1	Periodontitis S2	Periodontitis S3	Periodontitis (All Groups)
MMP-8 (ng/mL)	1677	4188 **	3469 *	3337 *	4079 **
MMP-9 (ng/mL)	3292	5617 *	16031 *	4144	5850 *
TIMP-1 (ng/mL)	6538	3861	3636 *	2254 ***	3446 **
MMP-8/TIMP-1	0.209	1.265 **	0.936 **	2.093 ***	1.265 ***
MMP-9/TIMP-1	0.484	1.776 **	3.164 ***	2.769 ***	2.296 ***

MMP: matrix metalloproteinase, TIMP: tissue inhibitor of metalloproteinase, S: stage, ^§^ Concentration (median), Significant difference at * *p* < 0.05, ** *p* < 0.01, *** *p* < 0.001 using Kruskal-Wallis and Bonferroni post hoc test of periodontitis groups as compared to healthy control.

**Table 3:** Diagnostic properties of statistically significant thresholds of examined biomarkers and combinations.

Biomarker	Sensitivity	Specificity	AUC	95% CI	Cut-Off Point	*p* Value
Control vs. periodontitis						
MMP-8 (ng/mL)	0.886	0.750	0.892	0.800 to 0.984	1992	0.0001
MMP-9 (ng/mL)	0.800	0.750	0.844	0.730 to 0.956	3606	0.002
TIMP-1 (ng/mL)	0.909	0.750	0.920	0.842 to 0.998	5994	0.0001
MMP-8/TIMP-1	0.977	1.000	0.986	0.956 to 1.000	0.464	0.0001
MMP-9/TIMP-1	1.000	1.000	1.000	1.000 to 1.000	0.712	0.0001
Control vs. periodontitis S1						
MMP-8 (ng/mL)	0.929	0.875	0.964	0.895 to 1.00	2190	0.0001
MMP-9 (ng/mL)	0.929	0.75	0.938	0.841 to 1.00	3606	0.001
TIMP-1 (ng/mL)	0.786	1.000	0.902	0.767 to 1.00	4734	0.002
MMP-8/TIMP-1	1.000	1.000	1.000	1.00 to 1.00	0.464	0.0001
MMP-9/TIMP-1	1.000	1.000	1.000	1.00 to 1.00	0.712	0.0001
S1 vs. S3						
TIMP-1 (ng/mL)	0.733	0.786	0.738	0.544 to 0.931	3228	0.029

MMP: matrix metalloproteinase, TIMP: tissue inhibitor of metalloproteinase, CI: confidence interval, S: stage.

## Data Availability

The data presented in this study are available from the corresponding author upon request.
